# Supplementary material for: Thymic dendritic cell-derived IL-27p28 promotes the establishment of functional bias against IFN-γ production in newly generated CD4+ T cells through STAT1-related epigenetic mechanisms
Source: eLife. 2025 May 14;13:RP96868. doi: 10.7554/eLife.96868 (PMC12077877; doi:10.7554/eLife.96868)
Supplement: Figure 1—source data 1. [file elife-96868-fig1-data1.zip › Figure 1 source data 1/Figure1-sourse data 1.pdf]

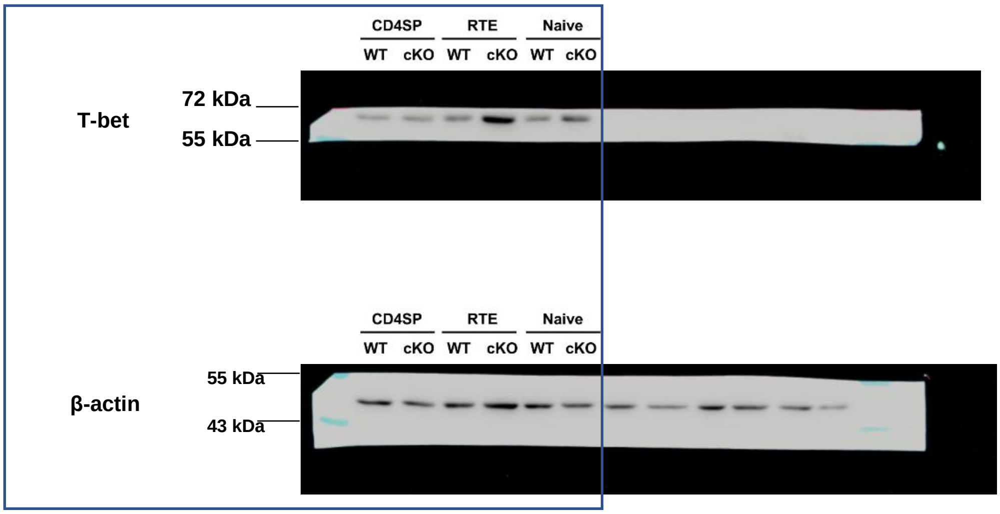

Figure 1 Source data 1 Original membranes corresponding to Figure 1E. The upper membranes correspond to T-bet, and the lower membranes correspond to β-actin. Rainbow molecular weight markers were employed. The areas highlighted by blue boxes are used in this result figure.
