# Supplementary figures and images for: Thymic dendritic cell-derived IL-27p28 promotes the establishment of functional bias against IFN-γ production in newly generated CD4+ T cells through STAT1-related epigenetic mechanisms
Source: eLife. 2025 May 14;13:RP96868. doi: 10.7554/eLife.96868 (PMC12077877; doi:10.7554/eLife.96868)

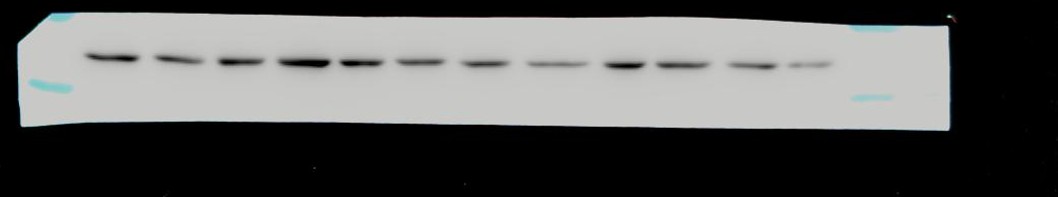

Supplement: Figure 1—source data 2. [file elife-96868-fig1-data2.zip › Figure 1 source data 2/Figure1E beta-actin.jpg]

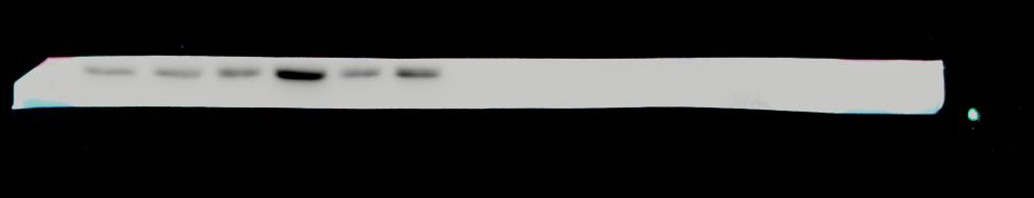

Supplement: Figure 1—source data 2. [file elife-96868-fig1-data2.zip › Figure 1 source data 2/Figure1E T-bet.jpg]

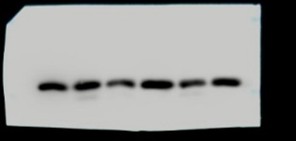

Supplement: Figure 3—figure supplement 1—source data 2. [file elife-96868-fig3-figsupp1-data2.zip › Figure 3 figure supplement 1 data2/H3.jpg]

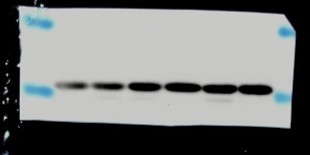

Supplement: Figure 3—figure supplement 1—source data 2. [file elife-96868-fig3-figsupp1-data2.zip › Figure 3 figure supplement 1 data2/H3K27me3.jpg]

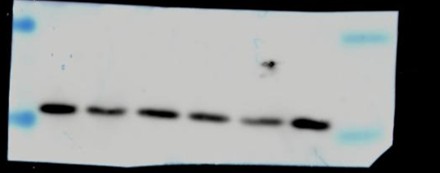

Supplement: Figure 3—figure supplement 1—source data 2. [file elife-96868-fig3-figsupp1-data2.zip › Figure 3 figure supplement 1 data2/H3K4me3.jpg]

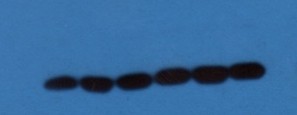

Supplement: Figure 3—figure supplement 1—source data 2. [file elife-96868-fig3-figsupp1-data2.zip › Figure 3 figure supplement 1 data2/H3K9me3.jpg]

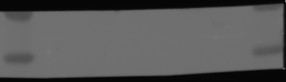

Supplement: Figure 5—source data 2. [file elife-96868-fig5-data2.zip › Figure 5 source data 2/beta-actin for STAT1 bf.jpg]

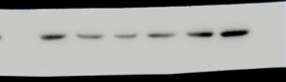

Supplement: Figure 5—source data 2. [file elife-96868-fig5-data2.zip › Figure 5 source data 2/beta-actin for STAT1.jpg]

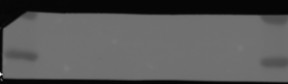

Supplement: Figure 5—source data 2. [file elife-96868-fig5-data2.zip › Figure 5 source data 2/beta-actin for STAT3 bf.jpg]

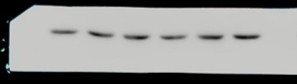

Supplement: Figure 5—source data 2. [file elife-96868-fig5-data2.zip › Figure 5 source data 2/beta-actin for STAT3.jpg]

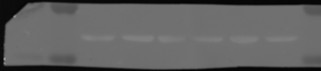

Supplement: Figure 5—source data 2. [file elife-96868-fig5-data2.zip › Figure 5 source data 2/beta-actin for STAT4 bf.jpg]

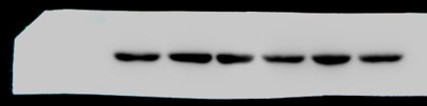

Supplement: Figure 5—source data 2. [file elife-96868-fig5-data2.zip › Figure 5 source data 2/beta-actin for STAT4.jpg]

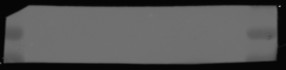

Supplement: Figure 5—source data 2. [file elife-96868-fig5-data2.zip › Figure 5 source data 2/p-STAT1 bf.jpg]

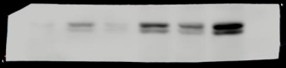

Supplement: Figure 5—source data 2. [file elife-96868-fig5-data2.zip › Figure 5 source data 2/p-STAT1.jpg]

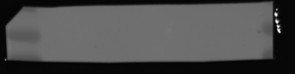

Supplement: Figure 5—source data 2. [file elife-96868-fig5-data2.zip › Figure 5 source data 2/p-STAT3 bf.jpg]

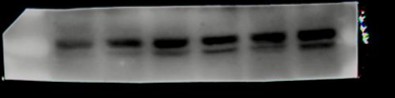

Supplement: Figure 5—source data 2. [file elife-96868-fig5-data2.zip › Figure 5 source data 2/p-STAT3.jpg]

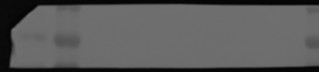

Supplement: Figure 5—source data 2. [file elife-96868-fig5-data2.zip › Figure 5 source data 2/p-STAT4 bf.jpg]

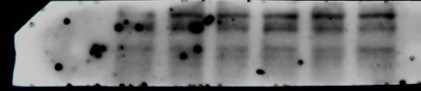

Supplement: Figure 5—source data 2. [file elife-96868-fig5-data2.zip › Figure 5 source data 2/p-STAT4.jpg]

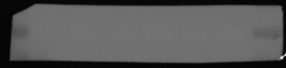

Supplement: Figure 5—source data 2. [file elife-96868-fig5-data2.zip › Figure 5 source data 2/STAT1 bf.jpg]

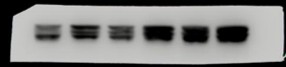

Supplement: Figure 5—source data 2. [file elife-96868-fig5-data2.zip › Figure 5 source data 2/STAT1.jpg]

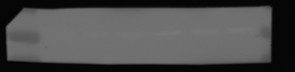

Supplement: Figure 5—source data 2. [file elife-96868-fig5-data2.zip › Figure 5 source data 2/STAT3 bf.jpg]

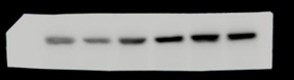

Supplement: Figure 5—source data 2. [file elife-96868-fig5-data2.zip › Figure 5 source data 2/STAT3.jpg]

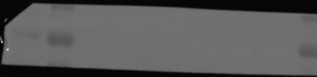

Supplement: Figure 5—source data 2. [file elife-96868-fig5-data2.zip › Figure 5 source data 2/STAT4 bf.jpg]

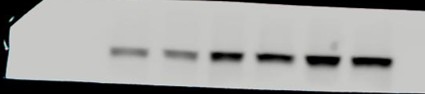

Supplement: Figure 5—source data 2. [file elife-96868-fig5-data2.zip › Figure 5 source data 2/STAT4.jpg]

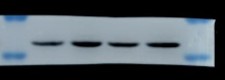

Supplement: Figure 5—figure supplement 1—source data 2. [file elife-96868-fig5-figsupp1-data2.zip › Figure5 figure supplement 1 data2/beta-actin.jpg]

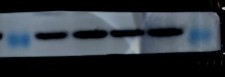

Supplement: Figure 5—figure supplement 1—source data 2. [file elife-96868-fig5-figsupp1-data2.zip › Figure5 figure supplement 1 data2/SOCS3.jpg]

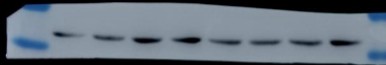

Supplement: Figure 5—figure supplement 2—source data 2. [file elife-96868-fig5-figsupp2-data2.zip › Figure 5 figure supplement 2 data2/beta-actin.jpg]

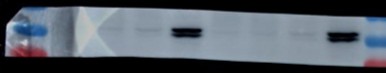

Supplement: Figure 5—figure supplement 2—source data 2. [file elife-96868-fig5-figsupp2-data2.zip › Figure 5 figure supplement 2 data2/p-STAT1.jpg]

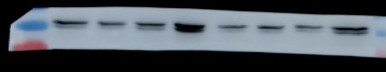

Supplement: Figure 5—figure supplement 2—source data 2. [file elife-96868-fig5-figsupp2-data2.zip › Figure 5 figure supplement 2 data2/STAT1.jpg]
