# Supplementary material for: Thymic dendritic cell-derived IL-27p28 promotes the establishment of functional bias against IFN-γ production in newly generated CD4+ T cells through STAT1-related epigenetic mechanisms
Source: eLife. 2025 May 14;13:RP96868. doi: 10.7554/eLife.96868 (PMC12077877; doi:10.7554/eLife.96868)
Supplement: Figure 3—figure supplement 1—source data 1. [file elife-96868-fig3-figsupp1-data1.zip › Figure 3 figure supplement 1 data1/Figure 3 figure supplement1-sourse data 1.pptx.pdf]

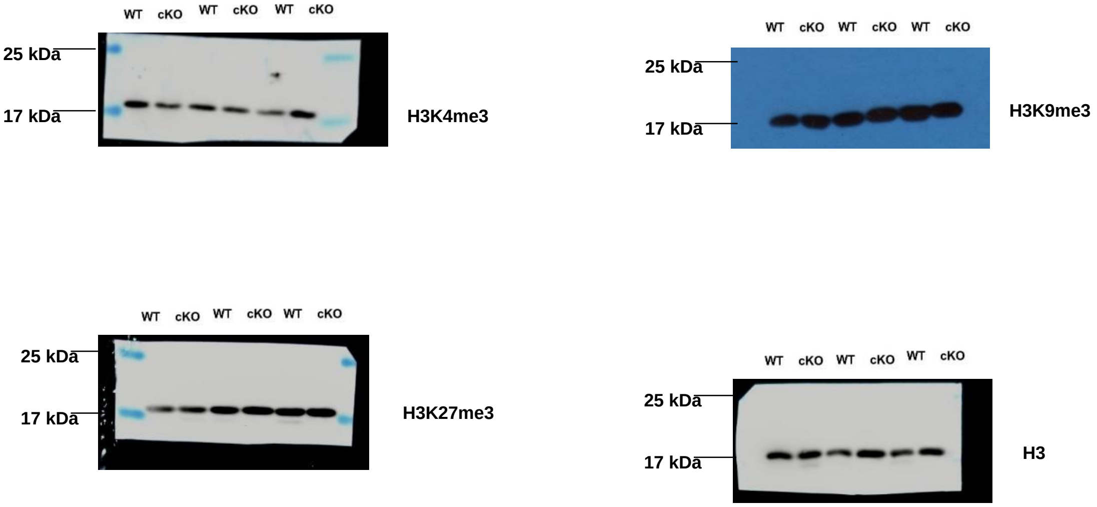

Figure 3-figure supplement 1 Original membranes corresponding to Figure 3-figure supplement 1A. The left two lanes are shown in the paper.
