# Supplementary material for: Thymic dendritic cell-derived IL-27p28 promotes the establishment of functional bias against IFN-γ production in newly generated CD4+ T cells through STAT1-related epigenetic mechanisms
Source: eLife. 2025 May 14;13:RP96868. doi: 10.7554/eLife.96868 (PMC12077877; doi:10.7554/eLife.96868)
Supplement: Figure 5—source data 1. [file elife-96868-fig5-data1.zip › Figure 5 source data 1/Figure 5-sourse data 1.pdf]

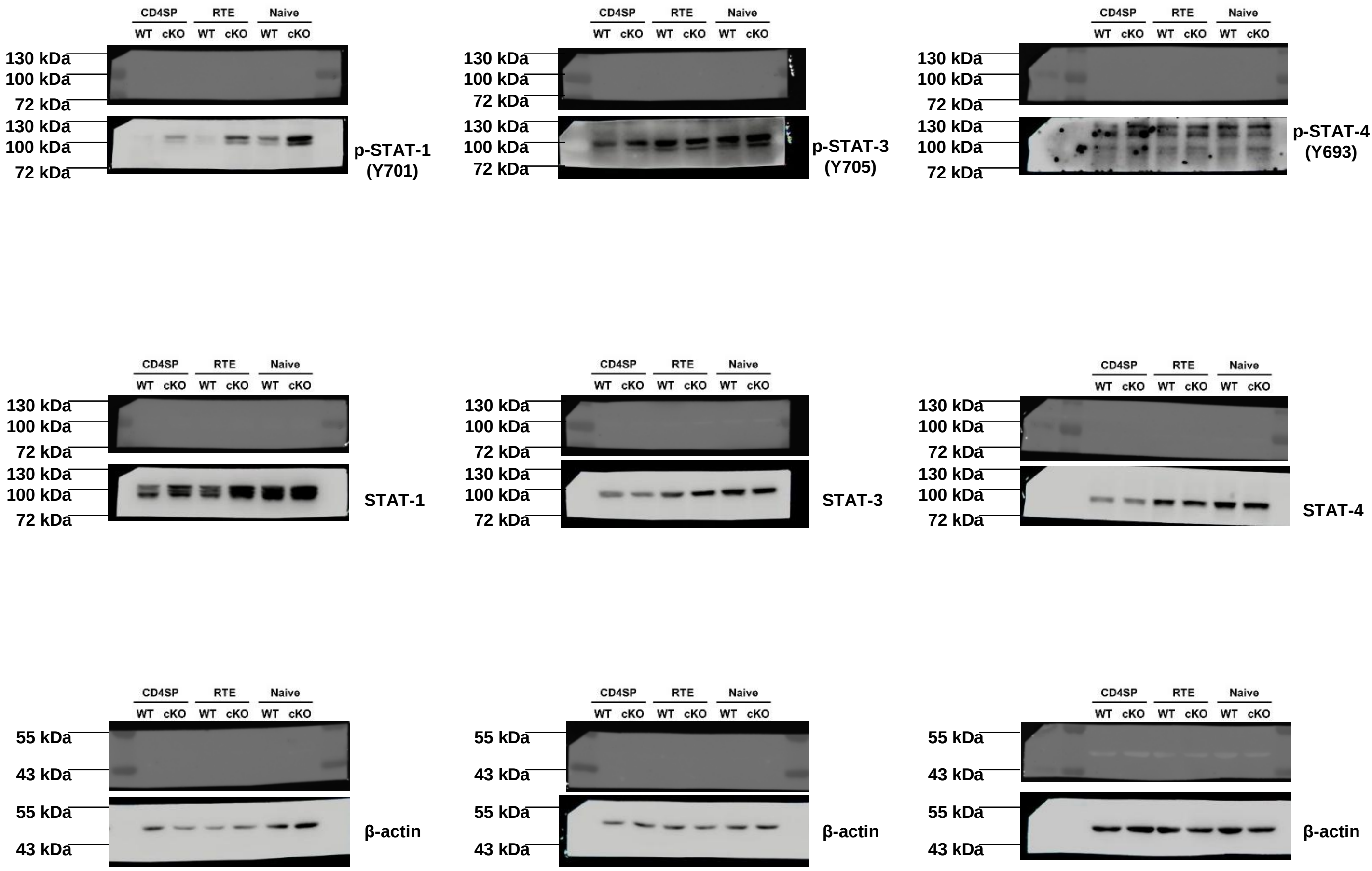

Figure 5 Source data 1 Original membranes corresponding to Figure 5B. The upper panel displays the rainbow molecular weight markers under bright-field imaging, while the lower panel corresponds to the bands of the detected molecules within the expected molecular weight range.
