# Supplementary material for: Thymic dendritic cell-derived IL-27p28 promotes the establishment of functional bias against IFN-γ production in newly generated CD4+ T cells through STAT1-related epigenetic mechanisms
Source: eLife. 2025 May 14;13:RP96868. doi: 10.7554/eLife.96868 (PMC12077877; doi:10.7554/eLife.96868)
Supplement: Figure 5—figure supplement 2—source data 1. [file elife-96868-fig5-figsupp2-data1.zip › Figure5 figure supplement 2 data1/Figure5 figure supplement2-sourse data 1.pdf]

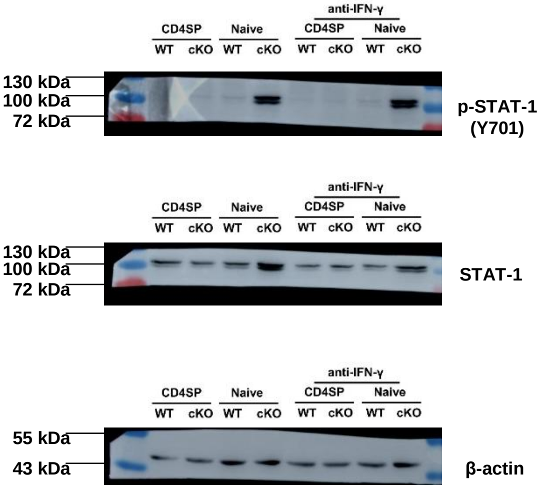

Figure 5-figure supplement 2 Original membranes corresponding to Figure 5-figure supplement 2. The upper membranes correspond to p-STAT1, the middle membranes correspond to STAT1, and the lower membranes correspond to β-actin. Rainbow molecular weight markers were employed.
