# Supplementary material for: Thymic dendritic cell-derived IL-27p28 promotes the establishment of functional bias against IFN-γ production in newly generated CD4+ T cells through STAT1-related epigenetic mechanisms
Source: eLife. 2025 May 14;13:RP96868. doi: 10.7554/eLife.96868 (PMC12077877; doi:10.7554/eLife.96868)
Supplement: Supplementary file 1. [file elife-96868-supp1.docx]

**Supplementary Table 1** Primers used in this study.

| Gene | Forward Primer | Reverse Primer |
| --- | --- | --- |
| *b-actin* | TATGGAATCCTGTGGCATC | GTGTTGGCATAGAGGTCTT |
| *Ifng* | TCAAGTGGCATAGATGTGGAAGAA | TGGCTCTGCAGGATTTTCATG |
| *Il4* | ACAGGAGAAGGGACGCCAT | GAAGCCCTACAGACGAGCTCA |
| *Il2* | TCCTGAGCAGGATGGAGAAT | GTCAAATCCAGAACATGCCG |
| *Tbx21* | TCAACCAGCACCAGACAGAG | ATCCTGTAATGGCTTGTGGG |
| *Gata3* | GCCTGCGGACTCTACCATAA | AGGATGTCCCTGCTCTCCTT |
| *Gm12250* | TAATGCCCTTCGGGGAATAGG | CTGGTTTGAAGTTAGTTGTCCCA |
| *Oasl2* | TTGTGCGGAGGATCAGGTACT | TGATGGTGTCGCAGTCTTTGA |
| *Usp18* | TTGGGCTCCTGAGGAAACC | CGATGTTGTGTAAACCAACCAGA |
| *Oas2* | TTGAAGAGGAATACATGCGGAAG | GGGTCTGCATTACTGGCACTT |
| *Oas3* | TCTGGGGTCGCTAAACATCAC | GATGACGAGTTCGACATCGGT |
| *Parp14* | AAGCAGATTGAAGTTGAGGACAA | CTTTGCCGGGGTTTCTGAAGT |
| *Ifit3* | TCAGGCTTACGTTGACAAGGT | CACACTTTAGGCGTGTCCATC |
| *Igtp* | CTCATCAGCCCGTGGTCTAAA | CACCGCCTTACCAATATCTTCAA |
| *Irf1* | ATGCCAATCACTCGAATGCG | TTGTATCGGCCTGTGTGAATG |
| *Ifi44* | AACTGACTGCTCGCAATAATGT | GTAACACAGCAATGCCTCTTGT |
| *Rsad2* | TGCTGGCTGAGAATAGCATTAGG | GCTGAGTGCTGTTCCCATCT |
| *Il12rb1* | ATGGCTGCTGCGTTGAGAA | AGCACTCATAGTCTGTCTTGGA |
| *Ifng-p* | CGTAATCCCGAGGAGCCTTC | CTTCAATGACTGTGCCGTGG |
| *Ifng-5.7* | AAAAAGAAAGTTCGCAGTCCC | CCTCGCTAGTCTGGCCAATAG |
| *Ifng+18* | TCACATGCCGACTAGTATAGGA | CTGAACTTCTAGGCGCTCAC |
| *Tbx21-p* | GAGGGTTGGTAGAGGATCAGAA | CTTACTTTTGAGATAAGATCCCCTGT |
| *Tbx21-9.6* | GGGACATCCGAGTCTCAGC | CTAGCCAATCCATTTTCATGC |
| *Tbx21+2.8* | GTGGTGGCACATCCTTGTAA | TCCTTTGAACTTCAGCTCTTCC |
| *Il4-p* | ACTCATTTTCCCTTGGTTTCAGC | GATTTTTGTCGCATCCGTGG |
| *Gata3+3* | GGGCGGATAGGTGGTAATG | ATGAAGCTGGAGACGTCTCA |
